# Supplementary material for: Content-rich biological network constructed by mining PubMed abstracts
Source: BMC Bioinformatics. 2004 Oct 8;5:147. doi: 10.1186/1471-2105-5-147 (PMC528731; doi:10.1186/1471-2105-5-147)
Supplement: Additional File 2 — The original results of the above study (non-essential files are deleted to keep the file size under the limit set by BMC bioinformatics). [file 1471-2105-5-147-S2.bz2 › chilibotAdditionalFile2/dip30/701ID2828368E18034/html/CYSN_CYSD.html]

 


 **CYSN** and **CYSD** 
  
Found 1720 abstracts in PubMed, retrieved 30.  
 

 What does Google say? 
 PDF only 
| .edu only 

---

**Interactive relationship** (e.g. stimulation, inhibition, etc)

**Inhibitory relationship**- The reactivity specific THMFP of post SAT  [ **CYSD** ]   [ **CYSN** ]  organic residuals with chlorine decreased slightly from pre SAT  [ **CYSD** ]   [ **CYSN** ]  levels 60 vs. 72& ; mgr ; g THM per mg DOC, respectively.  Ref: 12834733 Water Res< MedlineTA>Water Res,
- Compared with their healthy controls, SAT  [ **CYSD** ]   [ **CYSN** ]  Top of these PCOS O patients was strongly skewed into the android direction, providing significantly decreased leg SAT  [ **CYSD** ]   [ **CYSN** ]  development and significantly higher upper body obesity.  Ref: 12773703 Exp Biol Med Maywood , 2003
**Neutral relationship**- Among 84 consecutive stented patients, 16 patients who presented an SAT  [ **CYSD** ]   [ **CYSN** ]  group 3 were compared with 30 other stented patients free of SAT  [ **CYSD** ]   [ **CYSN** ]  group 4.  Ref: 12822144 Catheter Cardiovasc Interv, 2003
- SAT  [ **CYSD** ]   [ **CYSN** ]  2 is the serotype most often associated with outbreaks of foot and mouth disease FMD in livestock in southern and western Africa and is the only SAT  [ **CYSD** ]   [ **CYSN** ]  type to have been recorded outside the African continent in the last decade.  Ref: 12771430 J Gen Virol< MedlineTA>J Gen Virol, 2003

**Non-interactive relationship** (e.g. studied together, co-existance, homology, etc.)

- The first one was clinical symptoms such as fever, fatigue, arthralgia and generalized aches and the second was a titer of SAT  [ **CYSD** ]   [ **CYSN** ]  at least 1 160.  Ref: 12800954 Eur J Epidemiol, 2003
- The subjects then sat  [ **CYSD** ]   [ **CYSN** ]  outdoors for 10 minutes and their temperatures were again obtained time 10.  Ref: 12879387 Prehosp Emerg CarePrehosp Emerg Care, 2003
- Sat  [ **CYSD** ]   [ **CYSN** ]  P1 1 contained 335 nucleotides, and Sat  [ **CYSD** ]   [ **CYSN** ]  P1 2 contained 394 nucleotides.  Ref: 12765289 J Zhejiang Univ Sci< MedlineTA>J Zhejiang Univ Sci, 2003
- This difference appears to be related to the differing metabolic functions and secretory products of visceral adipose tissue VAT and subcutaneous adipose tissue SAT  [ **CYSD** ]   [ **CYSN** ] , as well as the fact that VAT drains directly into the liver.  Ref: 12793594 Int J Clin Pract Suppl, 2003
- We carried out a prospective evaluation of a new vasodilator stimulated phosphoprotein VASP phosphorylation assay in order to detect patients with high risk coronary subacute stent thrombosis SAT  [ **CYSD** ]   [ **CYSN** ]  despite thienopyridine regimen.  Ref: 12822144 Catheter Cardiovasc Interv, 2003
- Compared with healthy women, PCOS L patients had significantly lower total SAT  [ **CYSD** ]   [ **CYSN** ]  development even though height, weight, and body mass index did not deviate significantly, showing a slightly lowered amount of body fat in the upper region and a highly significant leg SAT  [ **CYSD** ]   [ **CYSN** ]  reduction.  Ref: 12773703 Exp Biol Med Maywood , 2003
- Furthermore, no significant differences were observed in the parameters of logistic models fitted to different strains within the serotypes A, O, and SAT  [ **CYSD** ]   [ **CYSN** ]  3, or when strains from serotypes A, O, and Asia 1, or SAT  [ **CYSD** ]   [ **CYSN** ]  1 and SAT  [ **CYSD** ]   [ **CYSN** ]  3, were combined.  Ref: 12804854 Vaccine, 2003
- Published reference values of 608 and 241 Italian healthy subjects were used as controls, respectively, for SF 36 and SAT  [ **CYSD** ]   [ **CYSN** ]  P.  Ref: 12823121 Allergy< MedlineTA>Allergy, 2003
- We therefore compared a silent substitution technique SST and a selective adaptation technique SAT  [ **CYSD** ]   [ **CYSN** ]  using a novel combination of optical filters, on 24 normal subjects.  Ref: 12828627 Ophthalmic Physiol Opt, 2003
- As regards patients with illness less than 3 months in duration, 93.1% tested positive by the dipstick assay, a percentage similar to that obtained in the standard serum agglutination test SAT  [ **CYSD** ]   [ **CYSN** ]  92.0%, somewhat lower than that obtained by culture 100% and higher than that obtained by IgM enzyme linked immunosorbent assay ELISA 80.5%.  Ref: 12853393 Clin Diagn Lab Immunol< MedlineTA>Clin Diagn Lab Immunol, 2003
- Furthermore, students who are in BSP have statistically lower high school grade point averages GPAs and Scholastic Achievement Test SAT  [ **CYSD** ]   [ **CYSN** ]  scores than students not in BSP.  Ref: 12888847 Cell Biol EducCell Biol Educ, 2003
- For comparison of the PCOS like body fat distribution with the healthy fat pattern, the previously published SAT  [ **CYSD** ]   [ **CYSN** ]  Top results of 590 healthy women and men 20 70 years old and 162 healthy girls and boys 7 11 years old were added to the factor plot.  Ref: 12773703 Exp Biol Med Maywood , 2003
- During infiltration, APEC and CAPEC concentrations decrease by more than 95% within 3m of SAT  [ **CYSD** ]   [ **CYSN** ] .  Ref: 12867334 Water Res, 2003
- SAT  [ **CYSD** ]   [ **CYSN** ]  P scores of CU patients compared with patients with respiratory allergy and with reference sample were significantly lower in many aspects of daily life.  Ref: 12823121 Allergy< MedlineTA>Allergy, 2003
- VASP phosphorylation analysis may be useful for the detection of coronary SAT  [ **CYSD** ]   [ **CYSN** ] .  Ref: 12822144 Catheter Cardiovasc Interv, 2003
- The SST method has already been shown to be highly sensitive in primary open angle glaucoma and, unlike SAT  [ **CYSD** ]   [ **CYSN** ] , has no significant age related variation.  Ref: 12828627 Ophthalmic Physiol Opt, 2003
- SAT  [ **CYSD** ]   [ **CYSN** ]  Top was examined in 33 women with polycystic ovary syndrome PCOS, in 87 age matched healthy controls and in 20 Type II diabetic women.  Ref: 12773703 Exp Biol Med Maywood , 2003
- Data regarding the expression of peroxisome proliferator activated receptor PPAR gamma 1 and PPAR gamma 2 in human visceral adipose tissue VAT and subcutaneous adipose tissue SAT  [ **CYSD** ]   [ **CYSN** ]  are conflicting.  Ref: 12829631 Diabetes< MedlineTA>Diabetes, 2003
- Based on the characterization techniques used, the character of bulk organics present in final SAT  [ **CYSD** ]   [ **CYSN** ]  water resembles the character of natural organic matter present in drinking water.  Ref: 12867327 Water ResWater Res, 2003
- STUDY DESIGN Intraexaminer and interexaminer procedure reliability and error analysis using a repeated measures design.OBJECTIVE To quantify sources of discrepancies in cervical range of motion values between two procedures that use the same potentiometric technology.SUMMARY OF BACKGROUND DATA Studies using an early version of an electrogoniometer system, which was connected between a helmet worn by the study participant and a chair on which they sat  [ **CYSD** ]   [ **CYSN** ] , reported unusually high values for active and passive cervical range of motion, although measurements were reliable.  Ref: 12811267 Spine, 2003
- The study revealed that final TOC concentrations of MF RO and SAT  [ **CYSD** ]   [ **CYSN** ]  are 0.3 and 1.0mgl 1, respectively.  Ref: 12867327 Water ResWater Res, 2003
- The cases n = 140 were defined by clinical symptoms and confirmed by a positive standard agglutination test SAT  [ **CYSD** ]   [ **CYSN** ] .  Ref: 12800954 Eur J Epidemiol, 2003
- Majority Asian and White students in BSP graduate with statistically similar UC GPAs despite having lower SAT  [ **CYSD** ]   [ **CYSN** ]  scores than non BSP majority students.  Ref: 12888847 Cell Biol EducCell Biol Educ, 2003
- MATERIALS AND METHODS Thirty five consecutive females with clinically suspected endometriosis underwent MRI examination using TSE T1, T2W, and SE FAT SAT  [ **CYSD** ]   [ **CYSN** ]  T1W sequences, to demonstrate the presence of endometriomas and pelvic implants.  Ref: 12835626 Radiol Med Torino , 2003
- We conclude that women with PCOS develop an android SAT  [ **CYSD** ]   [ **CYSN** ]  Top, but compared in more detail, we found two typical types of body fat distribution the childlike SAT  [ **CYSD** ]   [ **CYSN** ]  pattern in lean PCOS patients, and the diabetic body fat distribution in obese PCOS women.  Ref: 12773703 Exp Biol Med Maywood , 2003
- Biomass productivities up to 1.5 g l 1 per day and photosynthetic efficiency up to 14% were obtained by maintaining the cultures below 30 degrees C, dissolved oxygen levels less than 400% Sat  [ **CYSD** ]   [ **CYSN** ] .  Ref: 12814873 J Biotechnol< MedlineTA>J Biotechnol, 2003
- PCOS O women showed a SAT  [ **CYSD** ]   [ **CYSN** ]  Top pattern very similar to that of women with Type II diabetes, even though the diabetic women were on average 30 years older.  Ref: 12773703 Exp Biol Med Maywood , 2003
- Sat  [ **CYSD** ]   [ **CYSN** ]  P1 1 was identical to a satRNA Z96099 reported in 1995 in CMV P1.  Ref: 12765289 J Zhejiang Univ Sci< MedlineTA>J Zhejiang Univ Sci, 2003
- Fifteen anatomically well defined body sites from neck to calf describe the SAT  [ **CYSD** ]   [ **CYSN** ]  topography SAT  [ **CYSD** ]   [ **CYSN** ]  Top like an individual fingerprint.  Ref: 12773703 Exp Biol Med Maywood , 2003
- SAT  [ **CYSD** ]   [ **CYSN** ]  was the most sensitive test 87.0% for patients with illness more than 3 months in duration, followed by culture 50%, the dipstick assay 28.3%, and IgM ELISA 7.5%.  Ref: 12853393 Clin Diagn Lab Immunol< MedlineTA>Clin Diagn Lab Immunol, 2003
- The combined training group was separated into resistance training 3 days a week, Mon, Wed, Fri and the aerobic training 3 days a week, Tue, Thu, Sat  [ **CYSD** ]   [ **CYSN** ] .  Ref: 12808225 J Physiol Anthropol Appl Human Sci, 2003
- The new optical device, Lipometer, permits the noninvasive, quick, safe, and precise measurement of the thickness of subcutaneous adipose tissue SAT  [ **CYSD** ]   [ **CYSN** ]  layers at any given site of the human body.  Ref: 12773703 Exp Biol Med Maywood , 2003
- The attenuation of alkylphenol polyethoxylates APEOs metabolites was studied at a soil aquifer treatment SAT  [ **CYSD** ]   [ **CYSN** ]  site located in Arizona, USA.  Ref: 12867334 Water Res, 2003
- The male refractory period lasted 44.9 6.1s during which the male sat  [ **CYSD** ]   [ **CYSN** ]  and licked his genital region.  Ref: 12749942 Theriogenology, 2003
- RESULTS The SAT  [ **CYSD** ]   [ **CYSN** ]  and ST did not show statistically significant differences in both tests.  Ref: 12889705 Pediatr DentPediatr Dent, 2003
- Quantification of the expression of PPAR gamma 1 and PPAR gamma 2 in samples of VAT and SAT  [ **CYSD** ]   [ **CYSN** ]  was performed by real time RT PCR.  Ref: 12829631 Diabetes< MedlineTA>Diabetes, 2003
- However, only PPAR gamma 1 was differentially expressed, its levels in SAT  [ **CYSD** ]   [ **CYSN** ]  being 216 34% those in VAT P &lt.  Ref: 12829631 Diabetes< MedlineTA>Diabetes, 2003
- RESULTS Thirty medical students sat  [ **CYSD** ]   [ **CYSN** ]  both examinations and 20 returned evaluation questionnaires.  Ref: 12834416 Med Educ< MedlineTA>Med Educ, 2003
- We adopted two generic tools SF 36 an health status questionnaire and SAT  [ **CYSD** ]   [ **CYSN** ]  P a satisfaction profile.  Ref: 12823121 Allergy< MedlineTA>Allergy, 2003
- SF 36 and SAT  [ **CYSD** ]   [ **CYSN** ]  P scores of CU patients were compared with scores of a group of 27 patients with respiratory allergy.  Ref: 12823121 Allergy< MedlineTA>Allergy, 2003
- In both SAT  [ **CYSD** ]   [ **CYSN** ]  and VAT, the level of expression of PPAR gamma 2 were > ; 20 fold that of PPAR gamma 1 P &lt.  Ref: 12829631 Diabetes< MedlineTA>Diabetes, 2003
- S cone ERGs elicited by SAT  [ **CYSD** ]   [ **CYSN** ]  were of higher amplitude, p <.  Ref: 12828627 Ophthalmic Physiol Opt, 2003
- A 5 year program of study was conducted at the Sweetwater Recharge Facilities SRF to assess the performance of surface spreading operations for organics attenuation during field scale soil aquifer treatment SAT  [ **CYSD** ]   [ **CYSN** ]  of municipal wastewater.  Ref: 12834733 Water Res< MedlineTA>Water Res, 2003
- Diagnosis of brucellosis is made by a demonstration of high brucella agglutination titer using the Standard Tube Agglutination Test SAT  [ **CYSD** ]   [ **CYSN** ]  in the presence of compatible clinical symptoms.  Ref: 12797392 J Chemother, 2003
- Thus, it appears that increased VAT, but not SAT  [ **CYSD** ]   [ **CYSN** ] , is associated with both hepatic and peripheral biochemical abnormalities leading to insulin resistance and the associated metabolic syndrome.  Ref: 12793594 Int J Clin Pract Suppl, 2003
- Microfiltration MF followed by reverse osmosis RO and soil aquifer treatment SAT  [ **CYSD** ]   [ **CYSN** ]  are the two principal technologies considered for indirect potable reuse of wastewater.  Ref: 12867327 Water ResWater Res, 2003
- Both hikers started running for their lives when all of a sudden the CEO stopped, sat  [ **CYSD** ]   [ **CYSN** ]  down on a rock and started to put on running shoes.  Ref: 12886657 Mich Health HospMich Health Hosp, 2003
- Based on differences in the sequence and secondary structure between these two satRNAs, we conclude that Sat  [ **CYSD** ]   [ **CYSN** ]  P1 2 represents the emergence of a new satellite necrotic satellite from attenuated satRNA populations.  Ref: 12765289 J Zhejiang Univ Sci< MedlineTA>J Zhejiang Univ Sci, 2003
- However, the results of this study suggest that the new, SAT  [ **CYSD** ]   [ **CYSN** ]  method may prove to be more convenient and effective in clinical practice, provided that age norms are applied.  Ref: 12828627 Ophthalmic Physiol Opt, 2003
- METHODS A group of medical students sat  [ **CYSD** ]   [ **CYSN** ]  both the standard long case examination and the new objective structured clinical examination OSCE to introduce this latter examination to our Medical School for final MB.  Ref: 12834416 Med Educ< MedlineTA>Med Educ, 2003
- PURPOSE The purpose of this study was to evaluate a new simultaneous activation technique SAT  [ **CYSD** ]   [ **CYSN** ]  for the union between glass ionomer cements and composite resins.  Ref: 12889705 Pediatr DentPediatr Dent, 2003
- One hundred children with suspected congenital and or malformation and their parents who reported to SAT  [ **CYSD** ]   [ **CYSN** ]  hospital, Medical College, Trivandrum, India formed the test group.  Ref: 12884525 Genetika, 2003
- It is proposed that prior to metal insertion the metal may form a sitting atop SAT  [ **CYSD** ]   [ **CYSN** ]  complex with the invariant His Glu couple and the porphyrin.  Ref: 12761666 J Biol Inorg Chem, 2003
- CONCLUSIONS The SAT  [ **CYSD** ]   [ **CYSN** ]  is a less complex, quicker, and feasible alternative for the bonding of glass ionomer cements to composite resins in primary molars.  Ref: 12889705 Pediatr DentPediatr Dent, 2003
- In a stepwise, multivariate regression analysis, the levels of PPAR gamma 1 in both SAT  [ **CYSD** ]   [ **CYSN** ]  and VAT were the major determinants of waist circumference R 2 = 21% for both.  Ref: 12829631 Diabetes< MedlineTA>Diabetes, 2003
- In the SAT  [ **CYSD** ]   [ **CYSN** ]  treated effluent, EDTA and APECs were removed to approximately 4.3 and 0.54μg l, respectively, but were below the detection limit in the MF RO treated effluent.  Ref: 12867327 Water ResWater Res, 2003
- The implications of virus diversity within the SAT  [ **CYSD** ]   [ **CYSN** ]  2 serotype for control of foot and mouth disease in sub Saharan Africa.  Ref: 12771430 J Gen Virol< MedlineTA>J Gen Virol, 2003
- Ten male volunteers sat  [ **CYSD** ]   [ **CYSN** ]  in three postures erect, forward or anterior tilt, and posterior tilt.  Ref: 12763443 Clin Biomech Bristol, Avon , 2003
- Sequencing showed that the class 1 integron bearing strain contained two distinct gene cassettes, sat  [ **CYSD** ]   [ **CYSN** ]  1 and aadA.  Ref: 12890322 Emerg Infect Dis, 2003
- Sequence analysis showed there were two satRNAs Sat  [ **CYSD** ]   [ **CYSN** ]  P1 1 and Sat  [ **CYSD** ]   [ **CYSN** ]  P1 2.  Ref: 12765289 J Zhejiang Univ Sci< MedlineTA>J Zhejiang Univ Sci, 2003
- The hydrophilic most polar fraction of DOC was preferentially removed during SAT  [ **CYSD** ]   [ **CYSN** ] .  Ref: 12834733 Water Res< MedlineTA>Water Res, 2003
- This study provides an indication of the intratypic complexity of the SAT  [ **CYSD** ]   [ **CYSN** ]  2 serotype at the continental level and emphasizes the value of molecular characterization of diverse FMD field strains for tracing the origin of outbreaks.  Ref: 12771430 J Gen Virol< MedlineTA>J Gen Virol, 2003
- Two groups were restored with the SAT  [ **CYSD** ]   [ **CYSN** ]  and 2 by etching the ionomer surface sandwich technique ST.  Ref: 12889705 Pediatr DentPediatr Dent, 2003
- The BQS SAT  [ **CYSD** ]   [ **CYSN** ]  is designed to assess perceived barriers to quitting smoking among alcohol abusers using eleven true false items.  Ref: 12745034 J Subst Abuse Treat, 2003
- The Barriers to Quitting Smoking in Substance Abuse Treatment BQS SAT  [ **CYSD** ]   [ **CYSN** ]  was administered to 96 alcohol dependent smokers in residential substance abuse treatment.  Ref: 12745034 J Subst Abuse Treat, 2003
- SAT  [ **CYSD** ]   [ **CYSN** ]  Top differences of these three groups were described, and, based on a hierarchical cluster analysis, two distinctly different groups of PCOS women, a lean PCOS L and an obese PCOS O cluster, were found.  Ref: 12773703 Exp Biol Med Maywood , 2003
- This study, conducted at the Northwest Water Reclamation Plant, Mesa Arizona, evaluated MF RO and SAT  [ **CYSD** ]   [ **CYSN** ]  > ; 6 months residence time treated tertiary effluent with respect to organics removal.  Ref: 12867327 Water ResWater Res, 2003
